# The Association Between the Extent of Glioblastoma Resection and Survival in Light of MGMT Promoter Methylation in 326 Patients With Newly Diagnosed IDH-Wildtype Glioblastoma

**DOI:** 10.3389/fonc.2020.01087

**Published:** 2020-07-10

**Authors:** Fatih Incekara, Marion Smits, Sebastian R. van der Voort, Hendrik Jan Dubbink, Peggy N. Atmodimedjo, Johan M. Kros, Arnaud J. P. E. Vincent, Martin van den Bent

**Affiliations:** ^1^Department of Neurosurgery, Brain Tumor Center, Erasmus MC—University Medical Center Rotterdam, Rotterdam, Netherlands; ^2^Department of Radiology and Nuclear Medicine, Erasmus MC—University Medical Center Rotterdam, Rotterdam, Netherlands; ^3^Department of Pathology, Brain Tumor Center, Erasmus MC Cancer Institute, Rotterdam, Netherlands; ^4^Department of Neurology, Brain Tumor Center, Erasmus MC Cancer Institute, Rotterdam, Netherlands

**Keywords:** glioblastoma, extent of resection, contrast enhanced, non-contrast enhanced, survival, MGMT

## Abstract

**Background:** The association between contrast enhanced (CE) and non-contrast enhanced (NCE) tumor resection and survival in patients with glioblastoma in relation to molecular subtypes is poorly understood. The aim of this study was to assess the association between CE and NCE tumor resection and survival in light of MGMT promoter methylation in newly diagnosed IDH-wildtype glioblastoma.

**Materials and methods:** Patients with newly diagnosed IDH-wildtype glioblastoma who underwent surgery were eligible. CE and NCE tumor volumes were assessed on pre- and post-operative MRI scans and extent of resection was calculated. The association between CE and NCE tumor resection and survival was evaluated using multivariable Cox proportional hazards models and Kaplan Meier estimates.

**Results:** Three hundred and twenty-six patients were included: 177 (54.3%) with and 149 (45.7%) without MGMT methylation. Multivariable Cox proportional hazards models stratified for MGMT methylation identified age ≤ 65y (HR 0.63; 95% CI, 0.49–0.81; *p* < 0.0001), chemoradiation (HR 0.13; 95% CI, 0.09–0.19; *p* < 0.0001), maximal CE tumor resection (HR 0.58; 95% CI, 0.39–0.87; *p* = 0.009), ≥ 30% NCE tumor resection (HR 0.71; 95% CI, 0.53–0.93; *p* = 0.014), and minimal residual CE tumor volume (HR 0.64; 95% CI, 0.46–0.88 *p* = 0.007) as being associated with longer overall survival. Kaplan Meier estimates showed that extensive surgery was more beneficial for patients with MGMT methylated glioblastoma.

**Conclusions:** This study shows an association between maximal CE tumor resection, ≥30% NCE tumor resection, minimal residual CE tumor volume, and longer overall survival in patients with newly diagnosed IDH wildtype glioblastoma. Intraoperative imaging and stimulation mapping may be used to pursue safe and maximal resection. In future research, the safety aspect of maximizing tumor resection needs to be addressed.

## Introduction

Patients with glioblastoma have a poor prognosis with a median overall survival of 10–15 months, despite safe and maximal surgical resection followed by chemo- and radiotherapy (1). This prognosis varies based on known factors such as age, KPS, extent of resection, isocitrate dehydrogenase (IDH) mutation status, and methylguanine methyltransferase (MGMT) promoter methylation status (2, 3).

Maximal resection of the contrast enhanced (CE) portion of glioblastoma has been associated with better overall survival and is currently part of standard surgical glioblastoma treatment (3). However, glioblastoma is known to infiltrate far beyond the margins of CE as seen on MRI, into the surrounding non-contrast enhanced (NCE) edematous T2-weighted or FLAIR abnormality area (4). This raises the question whether maximal CE tumor resection should be extended beyond CE, into NCE area, to improve survival (5). A recent meta-analysis and a systematic review suggested that there is an association between maximal CE tumor resection with resection of NCE tumor and overall survival (5, 6). However, the quality of evidence of the available studies was low due to confounding and selection biases. On top, studies investigating the impact of CE and NCE resection have reported limited molecular data on IDH mutation and MGMT promoter methylation of their studied glioblastoma population (6, 7).

Thus, in light of the WHO 2016 reclassification, which now includes such molecular data, the association between CE and NCE glioblastoma resection and survival needs to be re-evaluated in a molecularly homogenous glioblastoma IDH-wildtype population, while considering MGMT promoter methylation (8). The aim of this study therefore, was to assess the association between CE and NCE tumor resection and survival in light of MGMT promoter methylation in a cohort of patients with newly diagnosed IDH-wildtype glioblastoma.

## Materials and Methods

### Patients

All consecutive patients aged 18 years or older, newly diagnosed with a CE mass lesion as seen on post-contrast T1-weighted MRI scans, histopathological confirmed as IDH-wildtype glioblastoma, who underwent tumor resection or biopsy between January 2012 and May 2018 at Erasmus Medical Center Rotterdam were considered for this retrospective study. Patients were eligible if pre- and immediate post-operative (<48 h) T2-weighted or FLAIR and post-contrast T1-weighted MRI scans were available together with complete molecular data on IDH mutation and MGMT methylation. Molecular analysis was *post-hoc* performed in patients with unknown IDH mutation or MGMT methylation status; patients without enough tumor material for molecular analysis or in whom assays failed to produce a test result were excluded. The study was approved by the Medical Ethical Committee of Erasmus Medical Center Rotterdam, who waived the need for written informed consent from the patients due to the retrospective nature of this study and the (emotional) burden that would result from contacting the patients or their relatives to obtain consent. The study was performed in accordance with the 1964 Helsinki Declaration and its later amendments or comparable ethical standards and reported following the Strengthening the Reporting of Observational Studies in Epidemiology (STROBE) guidelines.

### Image Acquisition, Tumor Segmentation, and Extent of Resection

From pre- and post-operative MRI scans, which were obtained in the clinical routine either on a 1.5T or 3.0T scanner, post-contrast T1-weighted and T2-weighted or FLAIR images were collected.

For glioblastoma segmentation, we imported both pre- and post-operative post-contrast T1-weighted and T2-weighted or FLAIR scans into Brainlab (BrainLab, Feldkirchen, Germany, version 2.1.0.15). Using the SmartBrush tool in Brainlab Elements, we semi-automatically segmented all tumor involved CE on pre-operative post-contrast T1-weighted scans (including the necrotic part, if present) and all tumor involved CE on post-operative post-contrast T1-weighted scans (excluding small vessels in the surgical cavity or hemorrhage). We then semi-automatically segmented all tumor-related NCE on both pre-and post-operative T2-weighted or FLAIR scans (excluding extra-lesional hemorrhage). We attempted to minimize the inclusion of surgery induced new T2-weighted or FLAIR abnormality by overlaying and carefully comparing pre- and post-operative MRIs. We manually corrected all segmentations when needed using the manual Brush tool. All tumor volumes were assessed while being blinded for patients' clinical outcome.

We finally obtained four tumor volumes (cm^3^): pre-operative and residual CE volumes and pre-operative and residual NCE volumes. We calculated the CE surrounding NCE volumes by subtracting CE volumes from the total NCE volumes. We calculated the extent of resection (EOR, %) separately for both the CE and NCE portion with the formula: [(pre-operative volume – residual volume)/pre-operative volume] ^*^ 100 (9). Maximal CE resection was categorized in our dataset as CE EOR >97%. NCE tumor resection was dichotomized into ≥30% resection and <30% resection based on threshold analyses (Figures S1, S2) and analyzed independently from CE tumor resection (i.e., irrespective of whether complete CE tumor resection was achieved or not). Tumors that were biopsied were segmented only on pre-operative MRI scans and their EOR was imputed as being 0%. In addition to the CE EOR categories (biopsy, subtotal, maximal resection), we categorized CE residual tumor volumes in >5, 1–5, and 0–1 cm^3^. While CE EOR is in part defined by CE residual tumor volume (as given by the above-mentioned EOR formula), the categories are not identical due to variations in pre-operative tumor volume.

### Molecular Analysis

Tumor tissue samples were obtained from patients through surgical resection or biopsy. Histopathological examination was performed by neuropathologists; IDH mutational analysis was assessed with Sanger sequencing or targeted next generation sequencing and MGMT methylation status was assessed with a methylation specific PCR, as described elsewhere (10–12).

### Statistical Analysis

Overall survival was defined as time from surgery to death (primary outcome) and progression-free survival (PFS) was defined as time from surgery till clinical or radiological progression (secondary outcome). Patients were censored at time of last clinical follow up date.

Statistical analyses were performed with SPSS 25.0 statistical software (IBM Corp.). Pre-operative and post-operative residual tumor volume distributions were skewed and therefore log transformed prior to statistical analysis. Descriptive statistics were tested between MGMT methylation status groups with the Chi Squared Test or Fisher Exact test in case of categorical variables, with the Kruskal Wallis test in case of continuous non-normal distributed data and with log rank tests to compare median overall survival and PFS when using Kaplan Meier analysis.

The associations between each variable and outcome were first tested with univariable Cox proportional hazards models and all variables with *p* < 0.10 (entry significance threshold) were selected for multivariable Cox proportional hazards models. These models were stratified for MGMT, because this variable violated the proportional hazards assumption. Hazard ratio (HR) with 95% Confidence interval (CI) was estimated for each variable within the model. A *p* < 0.05 was considered statistically significant.

## Results

There were 375 glioblastoma patients considered for this study. We excluded 36 (9.6%) due to insufficient tissue material for molecular analysis, and 13 (3.5%) because of the presence of IDH mutation. In total, 326 IDH-wildtype glioblastoma were included in our analysis: 177 (54.3%) with and 149 (45.7%) without MGMT promoter methylation. Maximal CE resection was achieved in 61 patients (18.7%), while in 187 (57.4%) patients maximal resection of CE could not be achieved. Seventy-eight patients (23.9%) underwent biopsy. In 156 (47.9%) patients, ≥30% NCE tumor resection was achieved and in 170 (52.1%) patients 0–30% NCE tumor resection was achieved. Median overall survival and PFS was 309 days (95% CI, 278–340) and 174 days (95% CI, 159–209), respectively. Further patient and tumor characteristics are presented in Table 1.

**Table 1:** Patient and tumor characteristics.

**Characteristics**			**MGMT promoter**				
	**All** ***n*** **%**		**Methylated** ***n*** **%**		**Unmethylated** ***n*** **%**		***p*-value**
	326	100	177	54.3	149	45.7	
**Sex**							**0.006**
Male	206	63.2	100	48.5	106	51.5	
Female	120	36.8	77	64.2	43	35.8	
**Age, Years**							0.122
≤ 65	162	49.7	81	50.0	81	50.0	
>65	164	50.3	96	58.5	68	41.5	
Mean, years (*SD*)	63.8 (10.5)		64.2 (10.9)		63.3 (10.1)		0.453
**KPS**							0.748
≤ 70	119	36.5	66	55.5	53	45.5	
> 70	207	73.5	111	53.6	96	46.4	
Mean (*SD*)	79.2 (12.4)	78.8 (12.2)	79.7 (12.6)	0.480			
**Pre-operative Tumor Volume, Median cm**^**3**^ **(IQR)**							
CE	34.9 (15.5–55.8)		30.7 (13.8–52.5)		39.9 (17.1–26.2)		0.050
NCE	72.1 (29.4–127.5)		78.1 (27.8–133.8)		64.9 (32.3–113.4)		0.249
**Residual Tumor Volume, Median cm**^**3**^ **(IQR)**							
CE	5.0 (1.41–12.3)		6.1 (1.8–12.5)		3.8 (1.1–12.0)		0.097
NCE	39.7 (18.3–73.7)		42.2 (17.1–79.6)		36.5 (20.4–64.2)		0.699
**Maximal CE Resection**							0.213
Yes	61	18.7	27	44.3	34	55.7	
No	187	57.4	105	56.2	82	43.8	
Biopsy	78	23.9	45	57.7	33	42.3	
Median EOR (IQR)	83.4 (14.2-94.8)		79.6 (0-91.9)		88.0 (25-7-96.0)		0.075
**NCE Resection**							0.609
≥30%	156	47.9	87	55.8	69	44.2	
<30%	170	52.1	90	52.9	80	47.1	
Median EOR (IQR)	27.1 (0–57.1)		28.7 (0–58.4)		25.8 (0–55.0)		0.948
**Adjuvant Therapy**							0.294
No therapy	61	18.7	38	62.3	23	37.7	
Radio- or chemotherapy alone	56	17.2	27	48.2	29	51.8	
Chemoradiation	209	64.1	112	58.4	97	41.6	
**Overall Patient Outcome**							
Median overall survival (95% CI)	309 (278.0–340.0)		334 (266.8–401.2)		305 (275.2-334.8)		**0.003**
Median progression-free survival (95% CI)	184 (159.3–208.7)		174 (131.2–216.8)		190 (162.0–218)		0.053

*MGMT, methylguanine methyltransferase; SD, standard deviation; CE, contrast enhancement; NCE, non contrast enhancement; EOR, extent of resection; IQR, Inter Quartile Range; CI, Confidence interval*.

Univariable Cox proportional hazards regression analysis identified age ≤ 65y (HR 0.64; 95% CI, 0.51–0.80; *p* < 0.0001), KPS >70 (HR 0.59; 95% CI, 0.47–0.74; *p* < 0.0001), MGMT promoter methylation (HR 0.72; 95% CI, 0.57–0.90; *p* < 0.004), adjuvant chemoradiation (HR 0.14; 95% CI, 0.11–0.19; *p* < 0.0001), smaller pre-operative CE tumor volumes (per cm^3^ HR 0.92; 95% CI, 0.83–1.01; *p* < 0.094), maximal CE tumor resection (HR 0.51; 95% CI, 0.36–0.72; *p* < 0.0001), and ≥30% NCE tumor resection (HR 0.72; 95% CI, 0.58–0.91; *p* = 0.005) as being associated with longer overall survival. As further presented in Table 2, the variables age ≤ 65y, KPS > 70, adjuvant chemoradiation, maximal CE resection, and ≥30% NCE tumor resection were also significantly associated with a longer PFS in univariable Cox regression analysis (*p* < 0.05). Kaplan Meier curves for overall survival and PFS for each variable are presented in Figure S3.

**Table 2:** Cox proportional hazards models for overall survival and progression-free survival.

**Variable**	**Median days, 95%CI**	**Overall survival**				**Median days, 95%CI**	**Progression-free survival**			
		**Univariable**		**Multivariable**			**Univariable**		**Multivariable**	
		**HR, 95% CI**	***P*-Value^**#**^**	**Adjusted HR, 95% CI**	***P*-Value^**§**^**		**HR, 95% CI**	***P*-Value^**#**^**	**Adjusted HR, 95% CI**	***P*-Value^**§**^**
**Age, years**										
>65	259, 191–327	^a^				144, 114–175	^a^			
≤ 65	358, 311–405	0.64, 0.51–0.80	**<0.0001**	0.63, 0.49–0.81	**<0.0001**	216, 181–251	0.70, 0.56–0.89	**0.003**	0.82, 0.64–1.06	0.123
**KPS**										
≤ 70	199, 142–256	^a^				120, 91–149	^a^			
>70	369, 319–419	0.59, 0.47–0.74	**<0.0001**	0.92, 0.71–1.19	0.545	223, 181–265	0.51, 0.40–0.65	**<0.0001**	0.59, 0.45–0.77	**<0.0001**
**MGMT Promoter**										
Unmethylated	305, 275–335	^a^		^b^		190, 162–218	^a^		^b^	
Methylated	334, 267–401	0.72, 0.57–0.90	**0.004**			174, 131–217	0.79, 0.62–1.00	**0.054**		
**Adjuvant Therapy**										
None	65, 46–84	^a^				47, 38–56	^a^			
Radiotherapy or chemotherapy	217, 146–288	0.38, 0.26–0.55	**<0.0001**	0.29, 0.19–0.43	**<0.0001**	129, 107–151	0.23, 0.15–0.36	**<0.0001**	0.22, 0.14–0.35	**<0.0001**
Chemoradiation	423, 367–479	0.14, 0.11–0.19	**<0.0001**	0.13, 0.09–0.19	**<0.0001**	254, 222–287	0.09, 0.06–0.13	**<0.0001**	0.09, 0.06–0.14	**<0.0001**
**Pre-operative CE Tumor Volume**										
smaller per cm^3^		0.92, 0.83–1.01	**0.094**	0.85, 0.75–0.95	**0.007**		0.94, 0.85–1.04	0.239	0.99, 0.88–1.11	0.860
**Maximal CE Resection**	^*^					^*^				
Biopsy	153, 106–200	^a^				100, 87–113	^a^			
No	309, 278–340	0.79, 0.60–1.04	**0.090**	0.87, 0.61–1.23	0.415	190, 170–210	0.80, 0.61–1.05	0.109	1.25, 0.89–1.81	0.228
Yes	441, 321–560	0.51, 0.36–0.72	**<0.0001**	0.58, 0.39–0.87	**0.009**	272, 210–334	0.59, 0.42–0.85	**0.004**	0.97, 0.63–1.50	0.898
**NCE Resection**	^*^					^*^				
<30%	266, 213–319	^a^				155, 104–206	^a^			
≥30%	344, 304–385	0.72, 0.58–0.91	**0.005**	0.71, 0.53–0.93	**0.014**	203, 166–240	0.82, 0.65–1.03	**0.087**	0.87, 0.65–1.15	0.325

a *Reference group*.

b *Proportional hazard assumption for MGMT was violated, therefore multivariable Cox regression model was stratified for MGMT*.

*HR, Hazard ratio; MGMT, methylguanine methyltransferase, CI Confidence Interval*.

**Figure 1:**
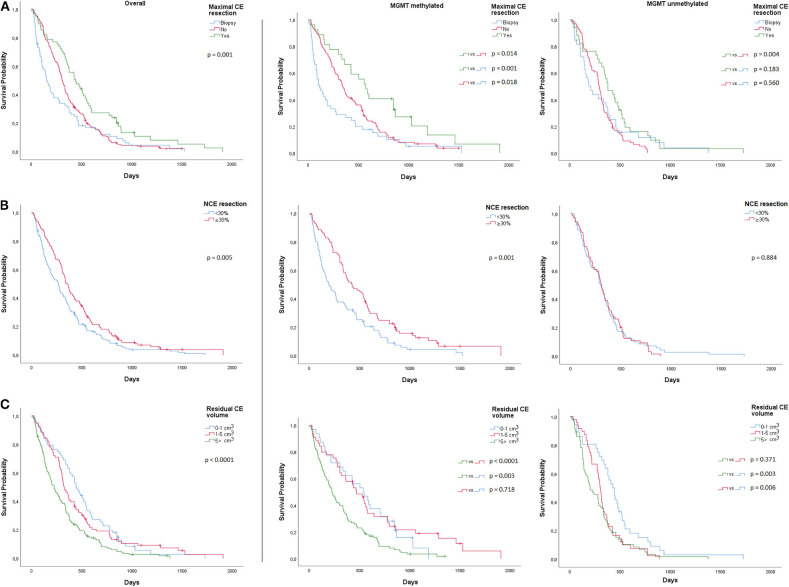
Overall and MGMT-stratified Kaplan Meier curves for overall survival **(A)** Maximal CE resection **(B)** NCE resection and **(C)** CE residual volume.

* *for MGMT-stratified median overall survival, see Figure 1*.

# *entry significance threshold of p < 0.1*.

§ *significance threshold of p < 0.05*.

Multivariable Cox proportional hazards regression analysis stratified for MGMT methylation status and risk adjusted for age ≤ 65y (HR 0.63; 95% CI, 0.49–0.81; *p* < 0.0001), KPS > 70 (HR 0.92; 95% CI, 0.71–1.19; *p* = 0.545), adjuvant chemoradiation (HR 0.13; 95% CI, 0.09–0.19; *p* < 0.0001), and smaller pre-operative CE tumor volumes per cm^3^ (HR 0.85; 95% CI, 0.75–0.95; *p* = 0.007) identified maximal CE tumor resection (HR 0.58; 95% CI, 0.39–0.87; *p* = 0.009) and ≥30% NCE tumor resection (HR 0.71; 95% CI, 0.53–0.93; *p* = 0.014) as being associated with longer overall survival (Table 2). Variables that remained significantly associated with a longer PFS were KPS > 70 (HR 0.59; 95% CI, 0.45–0.77; *p* < 0.0001) and adjuvant chemoradiation (HR 0.09; 95% CI, 0.06–0.14; *p* < 0.0001). Explorative multivariable Cox proportional hazards regression analyses showed that higher NCE tumor resection thresholds (e.g., ≥50%) were not associated with a favorable overall survival (*p* > 0.05) (for threshold analysis, see Figure S1).

Kaplan Meier estimates suggested that the impact of maximal CE tumor resection was more beneficial for patients with MGMT methylated glioblastoma and that it significantly improved median overall survival (572 days; 95% CI, 424–720), when compared to STR (342 days; 95% CI, 282–402; *p* = 0.014) or biopsy (112 days; 95% CI, 36–42; *p* = 0.001) (Figure 1A). Patients with MGMT methylated glioblastoma also had a longer overall survival with ≥30% NCE tumor resection (425 days; 95% CI, 286–564) than with <30% NCE tumor resection (190 days; 95% CI, 107–273; *p* = 0.001) (Figure 1B). In patients with MGMT unmethylated glioblastoma, no survival benefit was observed with ≥30% NCE resection (*p* = 0.884).

We further assessed minimal post-operative CE residual volumes with a potential positive impact on overall survival. In MGMT methylated glioblastoma, when compared to >5 cm^3^ residual CE tumor (224 days; 95% CI 164–284), we observed a significantly longer median overall survival for a residual CE tumor volume of 1–5 cm^3^ (470 days; 95% CI 330–610; *p* < 0.0001) and 0–1 cm^3^ (536 days; 95% CI 319–752; *p* = 0.003) (Figure 1C). In MGMT unmethylated glioblastoma, we only observed a longer overall survival in residual CE volumes of 0–1 cm^3^ (427 days; 95% CI, 350–503), when compared to 1–5cm^3^ (299 days; 95% CI, 270–327; *p* = 0.006) or to >5 cm^3^ (200 days; 95% CI, 102–298; *p* = 0.003) (Figure 1C). In these tumors, no difference in median overall survival was observed between 1–5 and >5 cm^3^ (*p* = 0.371) residual CE tumor volume. Multivariable Cox proportional hazards regression analysis stratified for MGMT and adjusted for age, KPS, adjuvant chemoradiation, and pre-operative CE tumor volume, identified a residual CE tumor volume of 0–1 cm^3^ (HR 0.64; 95% CI, 0.46–0.88 *p* = 0.007), and 1–5 cm^3^ (HR 0.71; 95% CI, 0.54–0.94; *p* = 0.016) as being associated with favorable overall survival. These analyses were also performed for post-operative residual NCE volumes, but here no minimal volume threshold with a positive impact on overall survival was identified.

## Discussion

This study shows an association between maximal CE tumor resection, ≥30% NCE tumor resection, minimal residual CE tumor volume and longer overall survival in 326 patients with newly diagnosed IDH-wildtype glioblastoma. We observed that extensive resection was more beneficial for patients with MGMT methylated IDH-wildtype glioblastoma in terms of survival.

Maximal resection of CE has earlier been associated with longer overall survival in a large meta-analysis of neurosurgical literature based on 37 studies and 41,117 unique glioblastoma patients (3). This association has recently been re-evaluated based on two new insights. First, studies performed before the WHO 2016 reclassification have included limited molecular data, because the impact of molecular subtyping of glioblastoma according to IDH mutation status was less of a consideration (3, 7, 13–15). Although IDH mutation within newly diagnosed primary glioblastoma is rare (<5 and 3.5% in our cohort) these tumors represent a distinct molecular type of glioma arising from a distinct precursor lesion (16, 17). Therefore, incomplete or absence of molecular data on IDH mutation and MGMT methylation or mixing molecular subtypes when evaluating the impact of glioblastoma resection on survival is undesirable. More recent studies did investigate the impact of glioblastoma surgery on survival in light of molecular markers. A recent study investigated the impact of maximal CE resection and MGMT promoter methylation status in a homogenous IDH-wildtype glioblastoma population (*n* = 175) and showed that both were significantly associated with longer overall survival (2). Ellingson et al. showed in 1,054 glioblastoma patients (with partially available data on IDH mutation and MGMT methylation) that smaller residual CE tumor volumes (<12 cm^3^) and MGMT methylation were significantly associated with longer overall survival in patients receiving chemoradiation (18). A recent study published by Molinaro et al. confirmed the association between maximal CE resection and overall survival across all molecular subgroups of glioblastoma (19).

Secondly, the association between glioblastoma resection and overall survival is also being reassessed by evaluating the value of NCE tumor resection, because it is known that glioblastoma infiltrates beyond the margins of CE into the NCE area (4). This aspect of glioblastoma surgery is also recently investigated by Molinaro et al. (19). The authors found that maximal resection of CE and NCE tumor was associated with longer overall survival in younger patients with IDH wildtype glioblastoma regardless of MGMT methylation status (subset of 190 patients with known IDH mutation and MGMT methylation status). In their study, maximal NCE resection that was associated with overall survival was defined as 92% NCE tumor resection after maximal CE tumor resection. Other studies have associated lower NCE tumor resection thresholds of 53% and 45% with overall survival (7, 20). We observed that a NCE tumor resection threshold of 30% was associated with overall survival. This association was studied CE tumor resection (i.e., irrespective of whether patients did or did not undergo complete CE tumor resection). In exploratory threshold analysis, higher thresholds (e.g., minimal ≥60% NCE tumor resection) seemed not to be associated with overall survival anymore. This may suggest that resection of NCE tumor immediately surrounding CE improves survival, but extending the resection on further distance from CE into NCE tumor does not provide survival benefit. It can be hypothesized that the direct peritumoral NCE area reflects a higher degree of tumor infiltration than the NCE area further away from the CE tumor, which is presumably more dominated by edema than tumor infiltration (4). In future research, a combination of physiological imaging modalities (such as MR spectroscopy, diffusion and perfusion imaging or positron-emission tomography) may be used to more accurately detect tumor infiltrated portions in NCE and to tailor surgical planning (21).

Importantly, maximal tumor resection should not be attempted at all cost. Previous studies assessing the value of extensive tumor resection generally reported no or only limited data on eloquent tumor location, neurological outcome or quality of patient life (3, 22, 23). The safety of extensive tumor resection is thus mostly unknown and should only be attempted with intraoperative imaging guidance and/or awake surgery with stimulation mapping, especially when tumors are closely related to eloquent brain area (24). While frontal or temporal pole tumor can commonly be removed more safely, posterior frontal or parietal tumors should be approached with more caution (25, 26).

In line with this, one limitation of this retrospective study is that we did not have sufficient data on eloquent tumor location or neurological outcome of patient after surgery. Therefore, the safety of extensive resection needs further investigation before clinical recommendations and the translation of potential survival benefits, into real world clinical practice can be made. Another limitation of this study is its retrospective nature. This may have introduced some degree of selection bias. We attempted to limit selection bias by consecutive inclusion of all glioblastoma patients operated upon between 2012 and 2018 in our cohort, including patients with complex glioblastoma localization (crossing midline or deep within the basal ganglia) who underwent diagnostic biopsies. We also performed IDH mutational and MGMT promoter methylation analyses on all glioblastoma included in our cohort. A third limitation is that only one observer assessed both pre- and post-operative tumor volumes. In this context, a stringent assessment of residual volumes in the resection cavity by one observer may explain the relatively low maximal CE resection percentage of 17.8%. Although the interobserver agreement is high for pre-operative volumes, it is known to be relatively low for residual tumor volumes. The intra-observer agreement nevertheless, is known to be high for both pre-operative as residual tumor volumes (9, 12). We have also attempted to limit bias during volumetric assessment by blinding the assessor for patients' clinical outcome. In future research, our findings need to be validated in an external validation. Ideally, the safety of maximizing tumor resection beyond CE tumor margins should be assessed in well-designed well-powered trials with safety measures as primary endpoint.

To conclude, this study shows an association between maximal CE tumor resection, ≥30% NCE tumor resection, minimal residual CE tumor volume, and longer overall survival in patients with newly diagnosed IDH wildtype glioblastoma. Extensive resection was more beneficial for patients with MGMT methylated glioblastoma. Intraoperative imaging and stimulation mapping may be used to pursue maximal tumor resection during glioblastoma surgery. In future research, the safety aspect of maximizing tumor resection needs to be addressed.

## Data Availability Statement

The raw data supporting the conclusions of this article will be made available by the authors, without undue reservation.

## Ethics Statement

The studies involving human participants were reviewed and approved by METC Erasmus MC (MEC-2019-0641). Written informed consent for participation was not required for this study in accordance with the national legislation and the institutional requirements.

## Author Contributions

FI, MS, and MB: literature search, study design, data collection, data analysis, data interpretation, and writing. SV, HD, PA, JK, and AV: data collection, data interpretation, and writing. All authors contributed to the article and approved the submitted version.

## Conflict of Interest

The authors declare that the research was conducted in the absence of any commercial or financial relationships that could be construed as a potential conflict of interest.

## Abbreviations

CE: contrast enhancement
IDH: isocitrate dehydrogenase
MGMT: O^6^-Methylguanine-DNA Methyltransferase
NCE: non-contrast enhancement.

## References

[1] Stupp R Mason WP van Den Bent MJ Weller M Fisher B Taphoorn MJB. Radiotherapy plus concomitant and adjuvant temozolomide for glioblastoma. New Engl J Med. (2005) 352:987–96. 10.1056/NEJMoa043330 15758009

[2] Gessler F Bernstock JD Braczynski A Lescher S Baumgarten P Harter PN. Surgery for glioblastoma in light of molecular markers: impact of resection and MGMT promoter methylation in newly diagnosed IDH-1 wild-type glioblastomas. Neurosurgery. (2019) 84:190–7. 10.1093/neuros/nyy049 29617848 PMC6500906

[3] Brown TJ Brennan MC Li M Church EW Brandmeir NJ Rakszawski KL. Association of the extent of resection with survival in glioblastoma: a systematic review and meta-analysis. JAMA Oncol. (2016) 2:1460–9. 10.1001/jamaoncol.2016.1373 27310651 PMC6438173

[4] Eidel O Burth S Neumann JO Kieslich PJ Sahm F Jungk C. Tumor infiltration in enhancing and non-enhancing parts of glioblastoma: a correlation with histopathology. PLoS One. (2017) 12:e0169292. 10.1371/journal.pone.0169292 28103256 PMC5245878

[5] de Leeuw CN Vogelbaum MA. Supratotal resection in glioma: a systematic review. Neuro Oncol. (2019) 21:179–88. 10.1093/neuonc/noy166 30321384 PMC6374756

[6] Incekara F Koene S Vincent A van den Bent MJ Smits M. Association between supratotal glioblastoma resection and patient survival: a systematic review and meta-analysis. World Neurosurg. (2019) 127:617–24.e2. 10.1016/j.wneu.2019.04.092 31004858

[7] Li YM Suki D Hess K Sawaya R. The influence of maximum safe resection of glioblastoma on survival in 1229 patients: can we do better than gross-total resection? J Neurosurg. (2016) 124:977–88. 10.3171/2015.5.JNS142087 26495941

[8] Louis DN Perry A Reifenberger G von Deimling A Figarella-Branger D Cavenee WK. The 2016 World Health Organization classification of tumors of the central nervous system: a summary. Acta Neuropathol. (2016) 131:803–20. 10.1007/s00401-016-1545-1 27157931

[9] Grabowski MM Recinos PF Nowacki AS Schroeder JL Angelov L Barnett GH. Residual tumor volume versus extent of resection: predictors of survival after surgery for glioblastoma. J Neurosurg. (2014) 121:1115–23. 10.3171/2014.7.JNS132449 25192475

[10] Dubbink HJ Atmodimedjo PN Kros JM French PJ Sanson M Idbaih A. Molecular classification of anaplastic oligodendroglioma using next-generation sequencing: a report of the prospective randomized EORTC brain tumor Group 26951 phase III trial. Neuro Oncol. (2016) 18:388–400. 10.1093/neuonc/nov182 26354927 PMC4767239

[11] Esteller M Garcia-Foncillas J Andion E Goodman SN Hidalgo OF Vanaclocha V. Inactivation of the DNA-repair gene MGMT and the clinical response of gliomas to alkylating agents. N Engl J Med. (2000) 343:1350–4. 10.1056/NEJM200011093431901 11070098

[12] Incekara F van der Voort S Dubbink HJ Atmodimedjo P Tewarie RN Lycklama G. Topographical mapping of 436 newly diagnosed IDH wildtype Glioblastoma with vs without MGMT promoter methylation. Front Oncol. (2020) 10:596. 10.3389/fonc.2020.00596 32477929 PMC7235346

[13] Stummer W Pichlmeier U Meinel T Wiestler OD. Fluorescence-guided surgery with 5-aminolevulinic acid for resection of malignant glioma: a randomised controlled multicentre phase III trial. Lancet Oncol. (2006) 7:392–401. 10.1016/S1470-2045(06)70665-9 16648043

[14] Senft C Bink A Franz K Vatter H Gasser T Seifert V. Intraoperative MRI guidance and extent of resection in glioma surgery: a randomised, controlled trial. Lancet Oncol. (2011) 12:997–1003. 10.1016/S1470-2045(11)70196-6 21868284

[15] Lacroix M Abi-Said D Fourney DR Gokaslan ZL Shi W DeMonte F. A multivariate analysis of 416 patients with glioblastoma multiforme: prognosis, extent of resection, and survival. J Neurosurg. (2001) 95:190–8. 10.3171/jns.2001.95.2.0190 11780887

[16] Lai A Kharbanda S Pope WB Tran A Solis OE Peale F. Evidence for sequenced molecular evolution of IDH1 mutant glioblastoma from a distinct cell of origin. J Clin Oncol. (2011) 29:4482-90. 10.1200/JCO.2010.33.8715 22025148 PMC3236649

[17] Yan H Parsons DW Jin G McLendon R Rasheed BA Yuan W. IDH1 and IDH2 mutations in gliomas. N Engl J Med. (2009) 360:765–73. 10.1056/NEJMoa0808710 19228619 PMC2820383

[18] Ellingson BM Abrey LE Nelson SJ Kaufmann TJ Garcia J Chinot O. Validation of postoperative residual contrast-enhancing tumor volume as an independent prognostic factor for overall survival in newly diagnosed glioblastoma. Neuro Oncol. (2018) 20:1240–50. 10.1093/neuonc/noy053 29660006 PMC6071654

[19] Molinaro AM Hervey-Jumper S Morshed RA Young J Han SJ Chunduru P. Association of maximal extent of resection of contrast-enhanced and non-contrast-enhanced tumor with survival within molecular subgroups of patients with newly diagnosed Glioblastoma. JAMA Oncol. (2020) 6:495–503. 10.1001/jamaoncol.2019.6143 32027343 PMC7042822

[20] Pessina F Navarria P Cozzi L Ascolese AM Simonelli M Santoro A. Maximize surgical resection beyond contrast-enhancing boundaries in newly diagnosed glioblastoma multiforme: is it useful and safe? A single institution retrospective experience. J Neuro-Oncol. (2017) 135:129–39. 10.1007/s11060-017-2559-9 28689368

[21] Verburg N Koopman T Yaqub MM Hoekstra OS Lammertsma AA Barkhof F. Improved detection of diffuse glioma infiltration with imaging combinations: a diagnostic accuracy study. Neuro Oncol. (2019) 22:412–422. 10.1093/neuonc/noz180 31550353 PMC7058442

[22] Jenkinson MD Barone DG Bryant A Vale L Bulbeck H Lawrie TA. Intraoperative imaging technology to maximise extent of resection for glioma review. Cochrane Database Syst Rev. (2018) 1:CD012788. 10.1002/14651858.CD012788.pub2 29355914 PMC6491323

[23] Sanai N Mirzadeh Z Berger MS. Functional outcome after language mapping for glioma resection. N Engl J Med. (2008) 358:18–27. 10.1056/NEJMoa067819 18172171

[24] Gogos AJ Young JS Morshed RA Hervey-Jumper SL. Berger MS. Awake Glioma surgery: technical evolution and Nuances. J Neurooncol. (2020) 147:515–24. 10.1007/s11060-020-03482-z 32270374

[25] Roh TH Kang SG Moon JH Sung KS Park HH Kim SH. Survival benefit of lobectomy over gross-total resection without lobectomy in cases of glioblastoma in the noneloquent area: a retrospective study. J Neurosurg. (2020) 132:895–901. 10.3171/2018.12.JNS182558 30835701

[26] Incekara F Smits M Vincent AJPE. Letter to the editor. Supratotal resection of glioblastoma. J Neurosurg. (2019) 16:1–2. 10.3171/2019.3.JNS19810 31419786

