# Supplementary figures and images for: The Association Between the Extent of Glioblastoma Resection and Survival in Light of MGMT Promoter Methylation in 326 Patients With Newly Diagnosed IDH-Wildtype Glioblastoma
Source: Front Oncol. 2020 Jul 10;10:1087. doi: 10.3389/fonc.2020.01087 (PMC7381265; doi:10.3389/fonc.2020.01087)

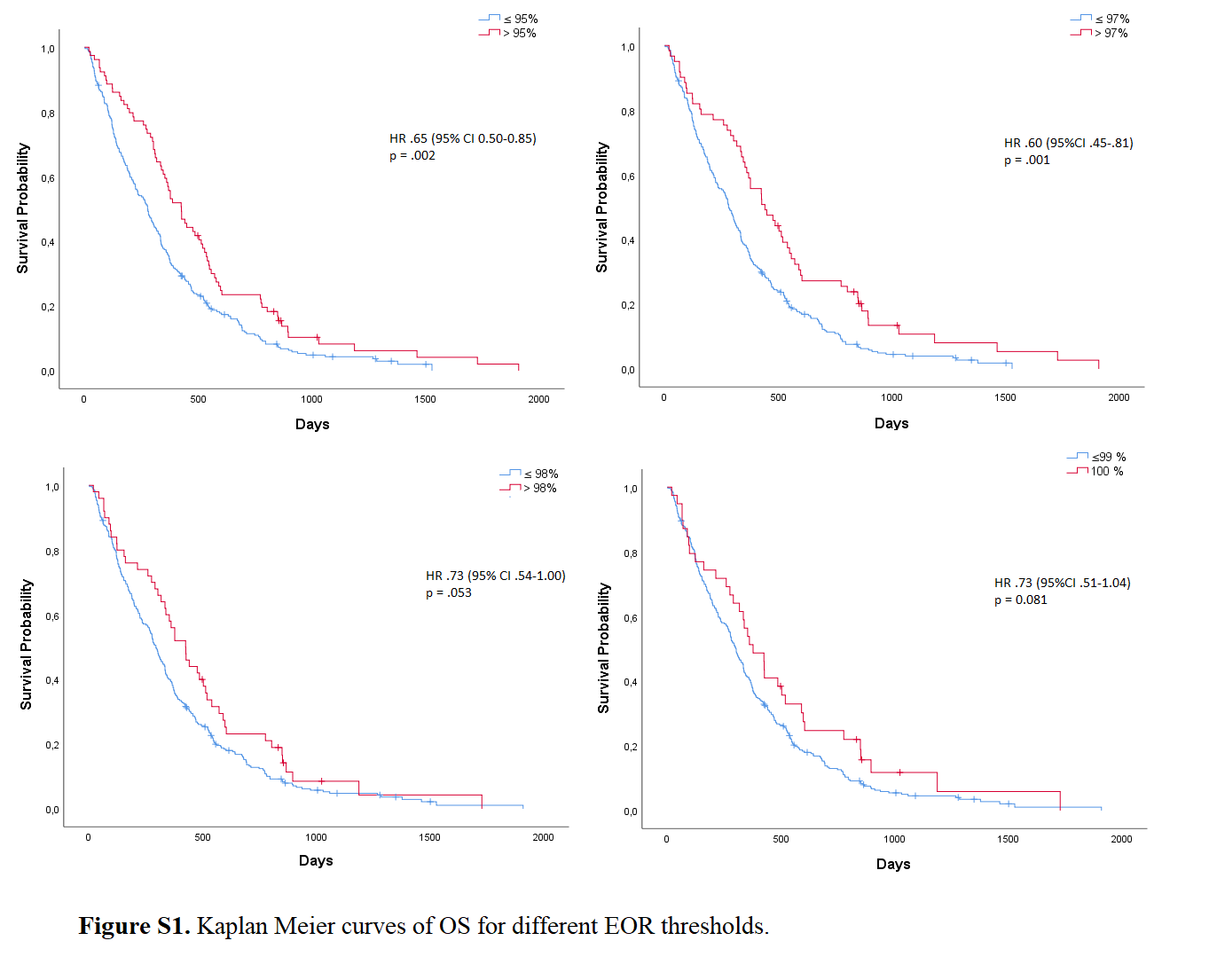

Supplement: Supplementary file 1 [file Image_1.tiff]

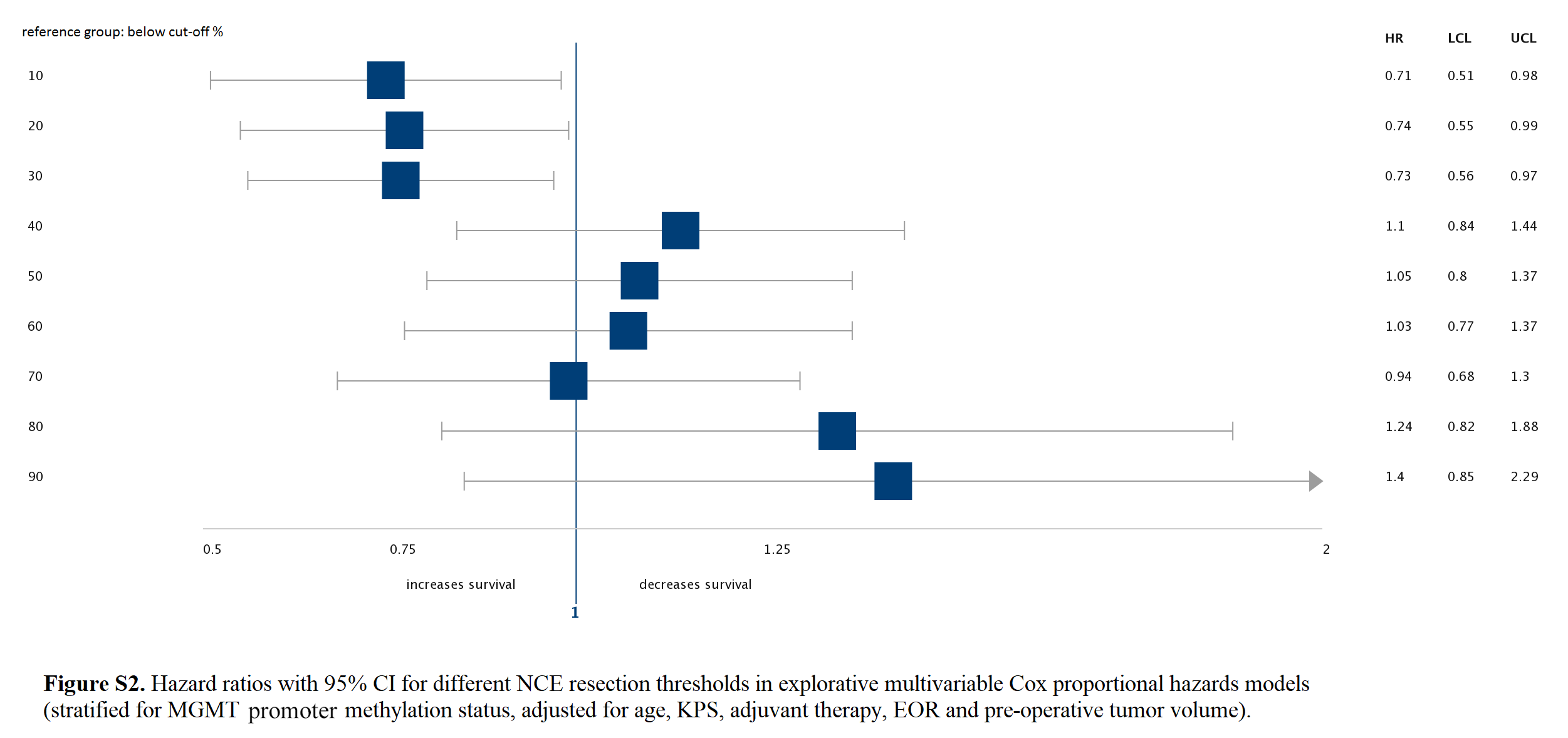

Supplement: Supplementary file 2 [file Image_2.tif]

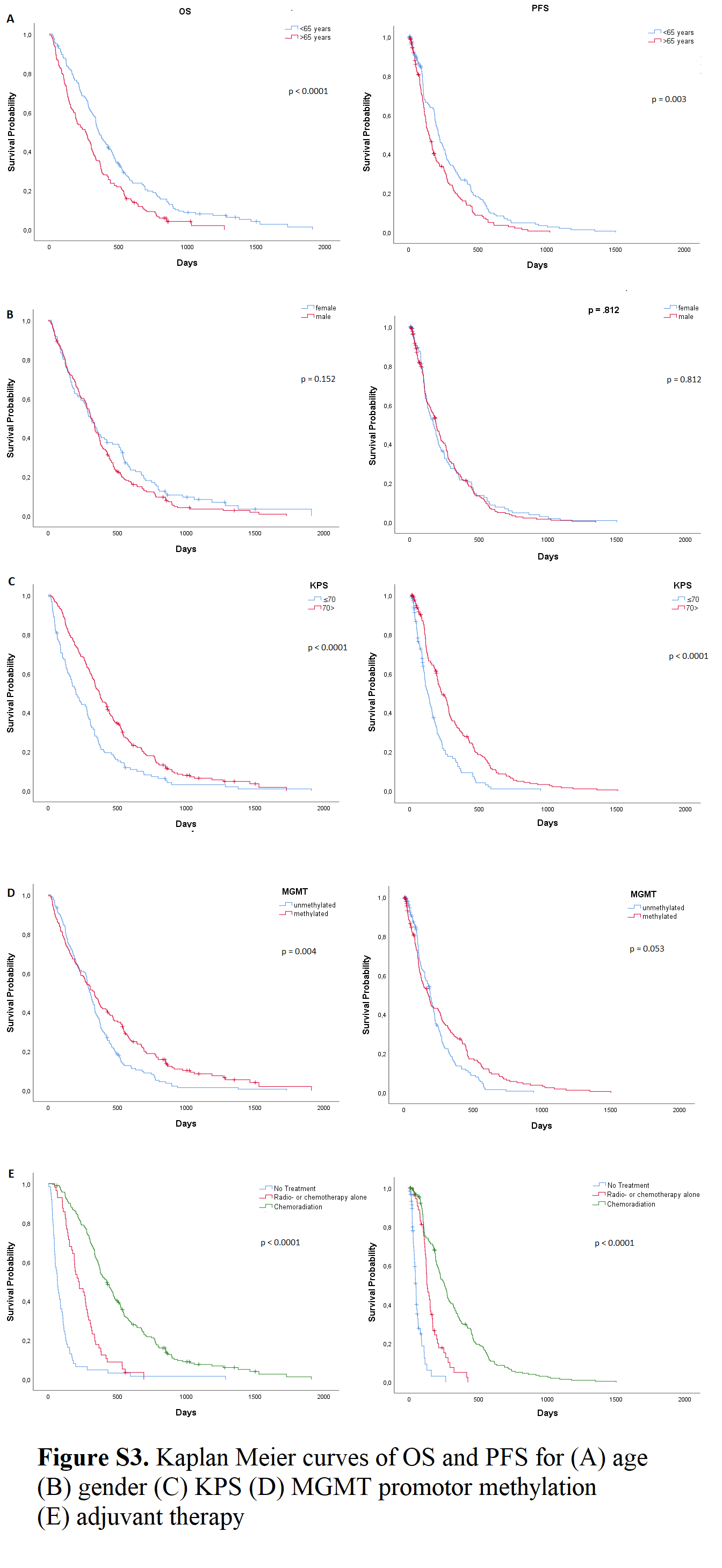

Supplement: Supplementary file 3 [file Image_3.tiff]
